# Association among extracellular superoxide dismutase genotype, plasma concentration, and comorbidity in the very old and centenarians

**DOI:** 10.1038/s41598-021-87982-6

**Published:** 2021-04-20

**Authors:** Takashi Sasaki, Yukiko Abe, Michiyo Takayama, Tetsuo Adachi, Hideyuki Okano, Nobuyoshi Hirose, Yasumichi Arai

**Affiliations:** 1 Center for Supercentenarian Medical Research, Keio University School of Medicine, 35 Shinanomachi, Shinjuku-ku, Tokyo, 160-8582 Japan; 2 Center for Preventive Medicine, Keio University School of Medicine, Tokyo, Japan; 3 Department of Biomedical Pharmaceutics, Laboratory of Clinical Pharmaceutics, Gifu Pharmaceutical University, Gifu, Japan; 4 Department of Physiology, Keio University School of Medicine, Tokyo, Japan

**Keywords:** Genotype, Clinical genetics, Biomarkers

## Abstract

Superoxide dismutase 3 (*SOD3*), an antioxidant enzyme, is known as extracellular SOD (EC-SOD) because it is the predominant form in extracellular fluids. The diversity of plasma EC-SOD concentration is associated with the *SOD3* p.R231G missense variant genotype. To clarify the association among *SOD3* genotype, plasma EC-SOD concentration, and comorbidity in Oldest Old, we analyzed genome-wide associations with plasma EC-SOD concentration and associations between EC-SOD concentration and medical history classified by the *SOD3* genotype in the Very Old (85–99 years old, n = 505) and Centenarians (over 100 years old, n = 595). The results revealed that *SOD3* p.R231G was the most significant variant associated with plasma EC-SOD concentration. Although no significant difference was observed in medical histories between the *SOD3* p.R231G variant non-carriers and carriers, higher EC-SOD concentration in plasma of *SOD3* p.R231G variant non-carriers was associated with a high odds ratio for chronic kidney disease (OR = 2.70, 95% CI = 1.98–3.72) and low odds ratio for diabetes mellitus (DM) (OR = 0.61, 95% CI = 0.39–0.95). Comparison with 11 plasma biomarkers for age-related disease showed that plasma EC-SOD concentration correlated with adiponectin and estimated glomerular filtration rate with creatinine correction; therefore, we deduced that EC-SOD co-operates with adiponectin and possesses beneficial functions for DM in the Oldest Old.

## Introduction

Aging is the process of getting older and it involves decline of several biological functions, which are thought to be associated with age-related diseases^1,2^. Superoxide is a monovalent anion (O_2_^−^) or a compound containing it, and excessive generation of superoxide and its related reactive oxygen molecules causes oxidative stress, which is thought to be one of the accelerating factors for aging^3^. Superoxide dismutase (SOD) is an antioxidant enzyme that catalyzes the dismutation of superoxide into hydrogen peroxide and oxygen molecules^4^. There are three isoforms of SOD genes, namely *SOD1*, *SOD2*,and *SOD3*, in mammals^5^. Among these SODs, *SOD3* is the predominant form in extracellular fluids such as plasma and binds to heparan sulfate proteoglycans and collagen, which anchors the SOD3 protein to the extracellular matrix; therefore, SOD3 protein is also called extracellular SOD (EC-SOD)^6,7^. Previous reports have revealed that the *SOD3* p.R231G variant (also called EC-SOD R213G) is located at the binding site for heparan sulfate proteoglycans, and this amino acid substitution to non-charged amino acids is expected to result in low affinity for heparan sulfate proteoglycans^8^. In fact, plasma EC-SOD concentration in humans was distributed as two discontinuous groups associated with the *SOD3* p.R231G variant^8–10^.

SODs have been reported to be associated with genetic diseases in humans. Mutations within *SOD1* have been implicated in human genetic diseases, such as amyotrophic lateral sclerosis^11^. Although *SOD3* is not a typical causal gene of Mendelian disease, the *SOD3* p.R231G variant has been variously reported as either a risk or protective genetic factor for the disease. Juul et al^12^ reported that the *SOD3* p.R231G variant was a risk factor for ischemic heart disease in 9,188 participants from the Copenhagen City Heart Study. Furthermore, the *SOD3* p.R231G variant was also reported to be associated with diabetes mellitus (DM) and several kinds of diabetes complications, including polyneuropathy, cardiovascular disease, myocardial infarction, and insulin resistance in DM patients^13–16^. Paradoxically, the *SOD3* p.R231G variant was found to be a protective genetic factor for chronic obstructive pulmonary disease and acute exacerbations of chronic obstructive pulmonary disease^17–19^. These reports suggest that the *SOD3* variant may be associated with age-related diseases by regulating EC-SOD in plasma.

To clarify the association among the SOD3 genotype, plasma EC-SOD concentration, and comorbidity in long-lived individuals who might have efficient anti-oxidant stress mechanisms, we screened single nucleotide variants (SNVs) associated with plasma EC-SOD concentration using a genome-wide association study (GWAS). We then analyzed the association between plasma EC-SOD concentration and medical history in the “Very Old” (85–99 years old) and “Centenarians” (over 100 years old). Finally, we classified the Very Old and Centenarians according to the genotype associated with plasma EC-SOD concentration and analyzed the association among plasma EC-SOD concentration, comorbidity, and biomarkers of age-related disease.

## Results

### Baseline characteristics of the very old and centenarians cohorts

We aggregated data from three prospective cohort studies: the Tokyo Centenarian Study (TCS), Japanese Semi-supercentenarian Study (JSS), and Tokyo Oldest Old Survey on Total Health (TOOTH)^20–24^. The analytic cohort comprised 1100 oldest-old individuals, including 505 Very Old (aged 85–99 years) and 595 Centenarians (100 years or older). The participants’ characteristics are presented in Table 1. The female percentages for the Very Old and Centenarians were 57.2 and 84.0%, respectively, and the median plasma EC-SOD concentration was 109.5 ng/µL and 147.4 ng/µL, respectively. Centenarians are characterized by a low prevalence of DM (DM, 6.1%) and a high prevalence of chronic kidney disease (CKD, 34.6%).

**Table 1:** Characteristics of participants according to age at enrollment.

	Very old			Centenarians		
	N	(85–99 years)		N	(100–111 years)	
Age at enrollment, median years (IQR)	505	87.7	(86.2–88.8)	595	104.9	(101.7–107.1)
Female, no. (%)	505	289	(57.2)	595	500	(84.0)
**Medical history**						
Diabetes Mellitus, no. (%)	505	79	(15.6)	583	36	(6.1)
Stroke, no. (%)	505	69	(13.6)	590	89	(15.0)
Coronary heart disease, no. (%)	505	43	(8.5)	590	87	(14.7)
Chronic kidney disease, no. (%)	505	73	(14.4)	595	206	(34.6)
Pneumonia, no. (%)	505	17	(3.3)	590	94	(15.9)
**Biomarkers in plasma**						
EC-SOD median, ng/mL (IQR)	505	109.5	(91.0–135.9)	595	147.4	(120.0–187.3 )
Adiponectin median, ng/mL (IQR)	505	12.1	(7.2–19.2)	594	18.5	(13.4–25.2)
Hemoglobin A1c median, %, (IQR)	505	5.9	(5.5–6.1)	587	5.6	(5.3–5.8)
NTproBNP median, ng/mL (IQR)	475	194.0	(115.5–392.0)	405	911.0	(450.0–1830.0)
eGFRcreat, median, mL/min/1.73m^2^ (IQR)	505	62.1	(52.2–71.3)	595	56.2	(39.0–66.5)
Cystatin C median, mg/dL (IQR)	503	1.17	(1.02–1.36)	593	1.60	(1.30–1.97)
TCHO median, mg/dL (IQR)	505	200.4	(178.0–221.0)	595	168.0	(143.0–189.5)
HDLC median, mg/dL (IQR)	505	58.2	(48.0–67.0)	595	48.6	(40.0–56.0)
LDLC median, mg/dL (IQR)	505	113.6	(95.0–130.0)	595	94.1	(74.0–112.5)
TNF-alpha median, pg/mL (IQR)	505	2.19	(1.87–2.79)	595	4.24	(3.30–5.55)
Interleukin-6 median, pg/mL (IQR)	505	1.66	(1.27–2.45)	595	3.15	(2.36–4.88)
CRP median, mg/dL (IQR)	505	0.090	(0.040–0.180)	595	0.180	(0.070–0.580)
**Genotype/sequencing**						
rs1799895 G allele, no. (MAF)	1010	56	(0.055)	1190	48	(0.040)
rs1799895 G allele carrier, no. (rate)	505	52	(0.102)	595	46	(0.077)
Whole genome sequenced, no. (%)	505	0	(0)	595	351	(58.9)

*IQR* interquartile range, *EC-SOD* extracellular superoxide dismutase, *NT-proBNP* N-terminal pro-brain natriuretic peptide, *eGFRcreat* estimated glomerular filtration rate based on serum creatinine, *TCHO* total cholesterol, *HDLC* high-density lipoprotein cholesterol, *LDLC* low-density lipoprotein cholesterol, *TNF-alpha* tumor necrosis factor-alpha, *CRP* C-reactive protein, *MAF* minor allele frequency.

### A genome-wide association study for plasma EC-SOD concentration in centenarians

**Figure 1:**
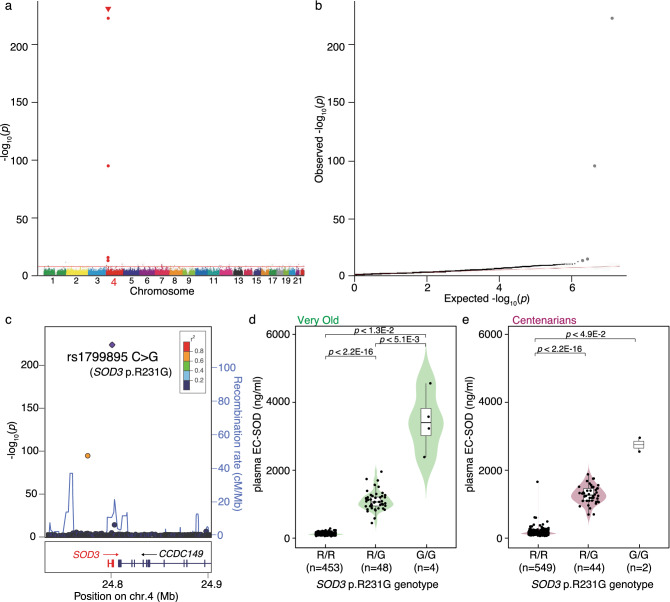
Plasma EC-SOD concentration-associated single nucleotide variants. (**a**) Manhattan plot of genome-wide association analysis of quantitative trait for plasma EC-SOD concentration. The most significant SNV was rs1799895, which corresponded to *SOD3* p.R231G missense variation (*p* = 2.18 × 10^−218^). Manhattan plot was drawn by qqman package in R script. (**b**) QQ-plot of genome-wide association analysis of quantitative trait for plasma EC-SOD concentration. This QQ plot indicates that *SOD3* p.R231G missense variation is strongly associated with plasma EC-SOD concentration. QQ-plot was drawn by qqman package in R script. (**c**) An enlarged view of a Manhattan plot around the *SOD3* gene. This enlarged view surrounding the *SOD3* gene indicates that the most significant SNV in the GWAS results associated with plasma EC-SOD concentration is located around the *SOD3* gene. This enlarged view of a Manhattan plot was drawn by LocusZoom. (**d**) Distribution of plasma EC-SOD concentration by *SOD3* p.R231G genotype in the Very Old, aged 85–99 years. Distribution of plasma EC-SOD concentration in the Very Old indicates that plasma EC-SOD concentration is dependent on the combination of the *SOD3* p.R231G genotype. (**e**) Distribution of plasma EC-SOD concentration by *SOD3* p.R231G genotype in Centenarians. Distribution of plasma EC-SOD concentration in the Centenarians indicates that plasma EC-SOD concentration depends on the combination of the *SOD3* p.R231G genotype.

It has been reported that the *SOD3*p.R231G missense variant in humans causes a reduction in the affinity for heparan sulfate proteoglycans in the extracellular matrix of endothelial cells, resulting in an increase in plasma EC-SOD concentration^8^. To confirm the genetic variants associated with plasma EC-SOD concentration, we analyzed SNVs through a GWAS with the quantitative traits associated with EC-SOD concentration in plasma using the whole-genome DNA sequences (WGS) of 351 Centenarians (Fig. 1a,b). The average number of reads for the WGS of 351 Centenarians was 1.08 million and the average read coverage depth was 40.5. The number of SNVs in which the minor allele frequency was more than 0.02 was 6,342,067.

From the results of the quantitative trait association analysis using EC-SOD concentration in plasma and 6,342,067 SNVs, rs1799895 C > G SNV, which corresponds to the *SOD3* p.R231G missense variant, was detected as the most significantly associated variant (*p* = 2.18 × 10^−218^). Although the other three variants were also detected as plasma EC-SOD concentration-associated variants, an enlarged view of the GWAS results indicated that these three variants were located around rs1799895 and would not be independent and weakly associated with rs1799895 (Fig. 1c). To confirm the relationship between the *SOD3* p.R231G genotype and plasma EC-SOD concentration, we genotyped rs1799895 in all Very Old and Centenarians and compared the distribution of plasma EC-SOD concentration among *SOD3* p.R231G genotypes (R/R (*SOD3* p.R231G variant non-carriers), R/G (*SOD3*p.R231G heterozygotes), and G/G (*SOD3*p.R231G homozygotes)) in the Very Old and Centenarians (Fig. 1d,e). From the results, the distribution of plasma EC-SOD concentration was significantly different depending on the *SOD3* p.R231G genotype in both the Very Old and Centenarians, except for one centenarian individual. These results indicate that plasma EC-SOD concentration could be determined based on the *SOD3*p.R231G genotype and an approximately tenfold difference in plasma EC-SOD concentration was observed between the *SOD3* p.R231G variant non-carriers and heterozygotes.

### *SOD3* p.R231G genotype is not associated with medical history in the Very Old and Centenarians

**Figure 2:**
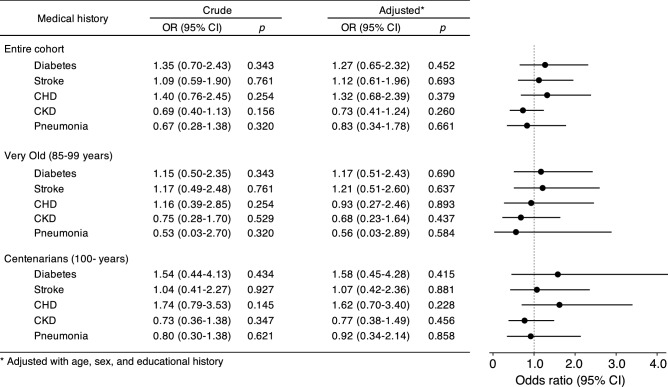
Odds ratios for medical history between the *SOD3* p.R231G non-carriers and heterozygotes. Number of *SOD3* p.R231G non-carriers and heterozygotes in Very Old was 453 and 48, respectively, and the number of *SOD3* p.R231G non-carriers and heterozygotes in Centenarians was 549 and 44, respectively. The results in the “Crude” column indicate the results analyzed using the generalized linear model without adjustment factor, and the results in the “Adjusted” column indicate the results analyzed using a generalized linear model adjusted with age, sex, and educational history. The black circles and lines in the figure indicate the odds ratio (OR) and range for 95% confidence interval (CI) in the “Adjusted” results, respectively. These results suggest that approximately a tenfold concentration difference in plasma EC-SOD by *SOD3* p.R231G genotypes was not associated with medical history, including DM, stroke, CHD, CKD, and pneumonia, in both the Very Old and Centenarians.

To evaluate the effect of the *SOD3* p.R231G genotype on comorbidity, we compared medical history including DM, stroke, coronary heart disease (CHD), CKD, and pneumonia among *SOD3* p.R231G genotypes between the *SOD3* p.R231G non-carriers and heterozygotes in the entire, Very Old, and Centenarians cohorts (Fig. 2). These results revealed that the medical history was mostly similar between the *SOD3* p.R231G non-carriers and heterozygotes in the entire, Very Old, and Centenarians, suggesting that the approximately tenfold difference in the plasma EC-SOD concentration among the *SOD3* p.R231G genotypes was not affected by medical history including DM, stroke, coronary heart CHD, CKD, and pneumonia in the Very Old and Centenarians.

### Association between plasma EC-SOD concentration and age by *SOD3* p.R231G genotype

**Figure 3:**
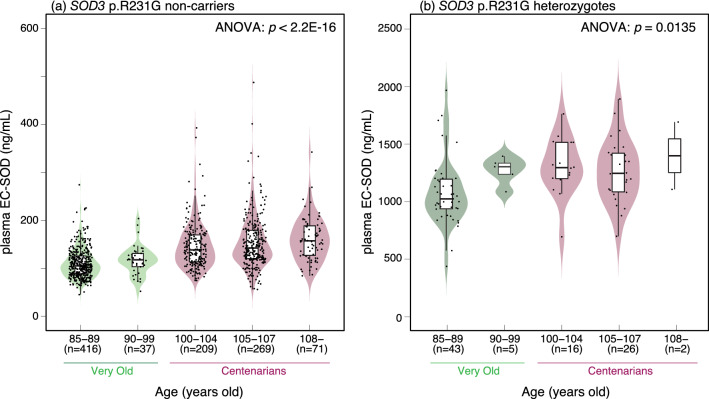
Distribution of plasma EC-SOD concentration by age. (**a**) Distribution of plasma EC-SOD concentration in *SOD3* p.R231G non-carriers by age. The distribution of plasma EC-SOD concentration classified by age showed a significant difference in *SOD3* p.R231G non-carriers (ANOVA: *p* < 2.2 × 10^−16^). (**b**) Distribution of plasma EC-SOD concentration in *SOD3* p.R231G heterozygotes by age. The distribution of plasma EC-SOD concentration classified by age showed a significant difference in *SOD3* p.R231G heterozygotes (ANOVA: *p* = 0.0135). These results suggest that the concentration of plasma EC-SOD increases depending on the age in the Very Old and Centenarian period.

As shown in Fig. 1d,e, the distribution range of plasma EC-SOD concentration in the *SOD3* p.R231G variant non-carriers showed a difference between the Very Old and Centenarian groups. To evaluate the difference in plasma EC-SOD concentration distribution in detail, we compared the distribution of plasma EC-SOD concentration by age in *SOD3* p.R231G variant non-carriers (Fig. 3a) and heterozygotes (Fig. 3b). The results showed that the distribution of the concentration of plasma EC-SOD by age was significantly different in both the *SOD3* p.R231G variant non-carriers (ANOVA: *p* < 2.2 × 10^−16^) and *SOD3* p.R231G heterozygotes (ANOVA: *p* = 0.0135). In the *SOD3* p.R231G variant non-carriers, the median of plasma EC-SOD concentration was gradually dependent on age, and the distribution range in the centenarians increased compared with that in the Very Old group, indicating that the plasma EC-SOD concentration in individuals with identical *SOD3* p.R231G genotypes would increase depending on the ages in the Very Old and Centenarian periods.

### Association between plasma EC-SOD concentration and medical history in *SOD3* p.R231G variant non-carriers in the Very Old and Centenarians

**Figure 4:**
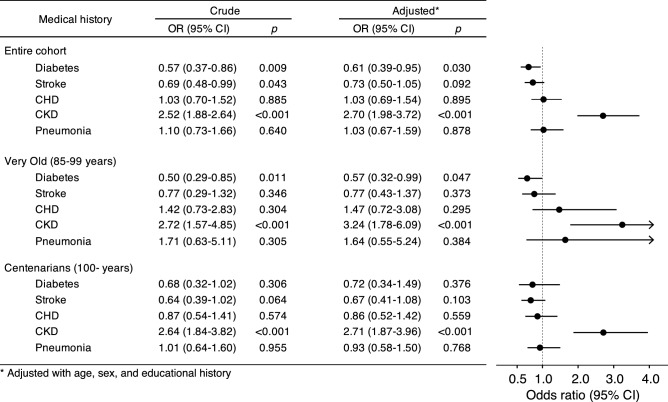
Odds ratios for medical history among top and bottom half groups of plasma EC-SOD concentration in *SOD3* p.R231G non-carriers in the Very Old and Centenarians. Total number of *SOD3* p.R231G non-carriers in Very Old and Centenarians was 453 and 549, respectively. The results in the “Crude” column indicate the results analyzed using the generalized linear model without adjustment factor and the results in the “Adjusted” column indicate the results analyzed using the generalized linear model adjusted with age, sex, and educational history. The black circles and lines in the figure indicate the odds ratio (OR) and range for 95% confidence interval (CI) in the “Adjusted” results, respectively. These results suggest that plasma EC-SOD concentration in the *SOD3* p.R231G variant non-carriers is associated with a high odds ratio for CKD in both the Very Old and Centenarians, while higher plasma EC-SOD concentration is only associated with low odds ratio for DM only in the Very Old.

To evaluate the association between plasma EC-SOD concentration and comorbidity in *SOD3* p.R231G variant non-carriers, we compared medical history including CKD, diabetes, stroke, CHD, and pneumonia among the top and bottom half groups of the EC-SOD concentration in *SOD3* p.R231G variant non-carriers of the entire cohort, Very Old, and Centenarians (Fig. 4). The results showed that a higher plasma EC-SOD concentration in the *SOD3* p.R231G variant non-carriers was associated with a high odds ratio for CKD in both the Very Old and Centenarians, while a higher plasma EC-SOD concentration was associated with a low odds ratio for DM only in the Very Old group. This indicates that the increment of plasma EC-SOD concentration for *SOD3* p.R231G variant non-carriers is associated with both DM and CKD in the Very Old group; however, this is only associated with CKD in the Centenarians.

### Association between plasma EC-SOD concentration and plasma biomarkers in *SOD3* p.R231G variant non-carriers

**Figure 5:**
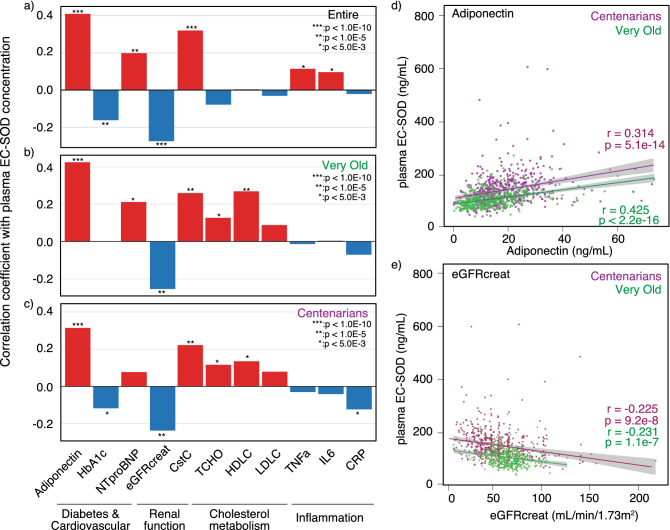
Correlation analysis of EC-SOD concentration with plasma biomarkers for age-related diseases. The bar charts indicate the correlation coefficient (r) between plasma EC-SOD and the concentrations of 11 plasma biomarkers for the entire cohort (**a**), the Very Old (**b**), and Centenarians (**c**). The red and blue bars indicate positive and negative correlations, respectively. The dot plots indicate the association between EC-SOD and adiponectin concentrations in plasma (**d**) and eGFRcreat (**e**) in the Very Old (green) and Centenarians (purple). Lines in the dot plot indicate correlation lines and the gray regions surrounding the correlation lines indicate the area for 95% confidence intervals.

The concentration of biomarkers in plasma is expected to provide important information on the pathophysiology of age-related diseases in the Oldest Old. To detect the plasma biomarkers associated with plasma EC-SOD concentration, we compared plasma EC-SOD concentration with plasma biomarkers for DM and cardiovascular disease (adiponectin and hemoglobin A1C [HbA1c], N-terminal pro-brain natriuretic peptide [NTproBNP]), renal function (cystatin C [CstC] and estimated glomerular filtration rate with creatinine correction [eGFRcreat]), cholesterol metabolism (total cholesterol [TCHO], high density lipoprotein cholesterol [HDLC], low-density lipoprotein cholesterol [LDLC]), and inflammation (tumor necrosis factor-alpha [TNF-α], interleukin-6 [IL-6], and C-reactive protein [CRP]) (Fig. 5a–c). The results revealed that the plasma EC-SOD concentration was correlated with the concentration of adiponectin in plasma (Fig. 5a–c, r = 0.41, *p* < 2.2 × 10^−16^; r = 0.43, *p* × 10^−16^; r = 0.31, *p* = 5.1 × 10^−14^ in the entire, Very Old, and Centenarians, respectively). Adiponectin is known to be one of the adipokines secreted from the adipose tissue and it possesses an antidiabetic effect; therefore, a higher adiponectin concentration associated with plasma EC-SOD concentration would result in a low prevalence of diabetes in *SOD3* p.R231G variant non-carriers of both the Very Old and Centenarians. In addition, we found a negative correlation between plasma EC-SOD concentration and eGFRcreat (Fig. 5a–c, r =  − 0.26, *p* < 2.2 × 10^−16^; r =  − 0.24, *p* = 1.1 × 10^−7^; r =  − 0.23, *p* = 9.2 × 10^−8^ in the entire, Very Old, and Centenarians, respectively), and a positive correlation between EC-SOD and CstC concentrations in plasma (Fig. 5a–c, r = 0.33, *p* < 2.2 × 10^−16^; r = 0.23, *p* = 6.8 × 10^−7^; r = 0.22, *p* = 3.8 × 10^−7^ in the entire, Very Old, and Centenarians, respectively). A detailed comparison of EC-SOD concentration, adiponectin, and eGFRcreat in plasma revealed that plasma EC-SOD concentration for Centenarians was higher than that for Very Old (Fig. 5d,e).

## Discussion

In this study, we analyzed the association between the genotypes corresponding to the *SOD3* p.R231G missense variant, plasma EC-SOD concentration, and comorbidity in both the Very Old and Centenarians. The *SOD3* p.R231G missense variant has a low affinity for heparan sulfate proteoglycans in the extracellular matrix of endothelial cells, resulting in two discontinuous groups for plasma EC-SOD concentration. A genome-wide association study of plasma EC-SOD concentration in Centenarians revealed that plasma EC-SOD concentration is associated with the genotype corresponding to the *SOD3* p.R231G missense variant; however, no other SNV associated with plasma EC-SOD concentration was isolated. These results suggest that the plasma EC-SOD concentration is mostly regulated by the *SOD3* p.R231G missense variant. Although previous studies have reported that the *SOD3* p.R231G genotype is associated with several diseases, including ischemic heart disease, DM, and DM-associated complications, our study revealed that approximately a tenfold difference in plasma EC-SOD concentration due to difference in the *SOD3* p.R231G genotype was not associated with the medical history of CKD, diabetes, stroke, CHD, and pneumonia in the Very Old and Centenarians^15,16^.

We expected that plasma EC-SOD concentration would be associated with not only the genotype of the *SOD3* p.R231G variant, but also with other genetic factors associated with binding between EC-SOD and heparan sulfate proteoglycans. We found 8 loci whose p-value was lower than GWAS significant level (5.0 × 10^−8^); however, no significant association was found between plasma EC-SOD concentration and combinational genotypes of these all 8 loci and *SOD3* p.R231G variant (data not shown). These associations on the GWAS were mainly observed when the homozygote of *SOD3* p.R231G variant was also a homozygote for SNVs with low frequency in Japanese cohort; therefore, we deduced that the combination of the genotypes including *SOD3* p.R231G and other low frequent variants would be a cause for “false” positive associations.

Plasma EC-SOD concentration increased with age in both the *SOD3* p.R231G variant non-carriers and heterozygotes, and it was correlated with adiponectin concentration in plasma in both the Very Old and Centenarians. These results suggested that the plasma EC-SOD concentration could be affected by non-genetic factors. We also found that a higher plasma EC-SOD concentration of the *SOD3* p.R231G variant non-carriers was associated with a low prevalence of DM in the Very Old group. It has been reported that both adiponectin and EC-SOD are secreted by adipocytes and that adiponectin plays a central role in obesity-related metabolic diseases, including type 2 diabetes^25–27^. Based on these results, we expected that the expression of both adiponectin and EC-SOD would increase in adipocytes with age, resulting in an increase in the concentration of both adiponectin and EC-SOD in plasma and a low prevalence of DM in the Very Old group. In the Centenarians, there was a relationship between EC-SOD and adiponectin concentrations in the plasma of *SOD3* p.R231G variant non-carriers; however, there was no relationship between higher plasma EC-SOD concentration and the prevalence of DM. The low prevalence of DM is one of the known characteristics of Centenarians; therefore, we deduced that other protective factors for DM may be affected by the prevalence of DM in Centenarians^28^.

In this study, we also revealed that a higher concentration of plasma EC-SOD is associated with a history of CKD and biomarkers for eGFRcreat in *SOD3* p.R231G variant non-carriers. Although the detailed mechanism underlying the association between CKD and higher concentration of plasma EC-SOD is still unknown, we deduced that clearance of proteins from the blood in patients with CKD was impaired, resulting in a high concentration of serum proteins including EC-SOD. A previous study reported that EC-SOD is normally expressed and localized in kidney tubules; however, localization of EC-SOD is significantly depleted in patients with fibrotic proteinuric CKD^29^. These results suggested the possibility that patients with CKD suffered from protein leakage from the kidney, which contains the EC-SOD protein.

Previous studies have reported mouse phenotypes of knock-in or transgenic mice for EC-SOD with R231G single nucleotide variation. The activity of EC-SOD did not change in knock-in mice with R231G single nucleotide variation; however, localization of EC-SOD protein was shifted, resulting in reduced susceptibility to lung disease and increased susceptibility to cardiopulmonary disease^30^. In humans, it is reported that the *SOD3* p.R231G variation leads to redistributing SOD3 protein from the lung parenchyma and vasculature into the plasma and epithelial lining fluid; therefore, the basic characteristics of EC-SOD were similar between humans and mice^30–32^. Kwon et al^33^ reported that transgenic mice with overexpression of SOD3_R213G_ (named SOD3_R231G_ in this study) exhibited premature aging, including hair graying, abnormal gait, and a shortened life span. We expected that *SOD3* p.R231G variation would not be associated with lifespan in humans because the minor allele frequency of *SOD3* p.R231G variation in both Very Old and Centenarians would not be significantly different from those of Japanese controls (data not shown)^34^. A detailed and careful phenotypic comparison is needed between humans and the SOD3_R213G_ transgenic mice because this comparison would be affected by both human-mouse differences and tissue-specific differences derived from the promoter of the transgene.

This study has several limitations. First, because of the cross-sectional design of the study, it was difficult to determine the causal relationship between plasma EC-SOD concentration and medical history. Another limitation was the small sample size of the genetic cohort study. In particular, the number of *SOD3* p.R231G variant carriers in the Very Old and Centenarians was 52 (MAF: 0.102) and 46 (MAF: 0.077), respectively. To reveal the causal relationship between plasma EC-SOD concentration and comorbidity, a longitudinal study with large sample sizes for both the Very Old and Centenarians is required.

In this study, we revealed the relationship among the *SOD3* p.R231G genotype, plasma EC-SOD concentration, and medical history in the Very Old and Centenarians. The detailed function of EC-SOD in plasma and the mechanism associated with medical history including DM and CKD have not been clearly elucidated; however, this study revealed that EC-SOD could co-operate and possess beneficial functions in the metabolic pathways with other biomarkers, including adiponectin, in older adults aged 85 years or older. Further research will shed light on the role of EC-SOD in achieving healthy longevity.

## Methods

### Study populations

We used data from three prospective cohort studies of elderly individuals in Japan: the Tokyo Centenarian Study (TCS)^20,21^, Japanese Semi-supercentenarian Study (JSS)^22,23^, and Tokyo Oldest Old Survey on Total Health (TOOTH)^24^. Recruitment was conducted as previously described. From the participants in both TCS and JSS, 595 centenarians (95 men, 500 women, mean age: 104.9 (IQR: 101.7–107.1)) with measured plasma EC-SOD concentration were enrolled in this study as “Centenarians” (Table 1).

The TOOTH survey was a community-based prospective cohort study of individuals aged between 85 and 102 years. In the TOOTH study, 542 individuals (236 men and 306 women) participated in medical and dental examinations. Of these, 505 individuals [216 men, 289 women, mean age: 87.7 (IQR: 86.2–88.8)] who are less than 100 and had measured plasma EC-SOD concentration were enrolled in this study as “Very Old.”

Written informed consent to participate in this study was obtained either from the participants or from their proxy when the person could not provide consent. All cohort studies were approved by the ethics committee of the Keio University School of Medicine (ID: 20021020, 20022020, 20070047) and are registered in the University Hospital Medical Information Network Clinical Trial Registry as observational studies (ID: UMIN000040446, UMIN000040447, UMIN000001842). All analyses in this study were conducted in accordance with the ethical guidelines for human genome/gene analysis research established by the Ministry of Education, Culture, Sports, Science and Technology, Ministry of Health, Labour and Welfare, and the Ministry of Economy, Trade and Industry in Japan.

### Baseline examination

All participants were examined directly by experienced geriatricians at the time of enrolment, in accordance with previously described protocols^20–24^. Our assessment protocols included basic personal information, medical history, and blood sampling.

### Biomarkers in blood

The concentration of blood biomarkers, including EC-SOD, adiponectin, NTproBNP, cystatin C (CstC), TNF-alpha, and interleukin-6 (IL-6) was measured in accordance with previously described protocols^35^. Blood test results for HbA1c, estimated glomerular filtration rate with creatinine correction (eGFRcreat), total cholesterol (TCHO), high-density lipoprotein cholesterol (HDLC), low-density lipoprotein cholesterol (LDLC), and C-reactive protein (CRP) were also obtained in accordance with previously described protocols^35^.

### Whole-genome DNA sequencing, mapping, and variant call

Total genomic DNA was extracted from whole blood using a FlexGene DNA Kit (QIAGEN, Hilden, Germany). A genomic DNA library for DNA sequencing for next-generation DNA sequencer was constructed using the Illumina DNA PCR-Free Prep kit (Illumina). The constructed genomic DNA library was sequenced using the HiSeq2500 and HiSeqX systems (Illumina). Finally, we obtained whole-genome DNA sequence data from 351 centenarians. A set of raw DNA sequence data (fastq format) was mapped into the hs37d5 reference genome DNA sequence using the bwa program (version 0.7.16), and a BAM format file was created using the GATK Best Practices Workflow with GATK version 3.7^36,37^. After applying the Variant Quality Score Recalibration (VQSR) program, the variant data for each individual were called using the HaplotypeCaller program in GVCF mode. After filtering all the INDELs and SNVs with a minor allele frequency (MAF) of less than 0.02, joint genotyping data including 6,342,067 variants were obtained using the GenotypeGVCFs program in GATK package^37^.

### Quantitative trait association analysis

To identify plasma EC-SOD concentration-associated SNVs, we analyzed data for plasma EC-SOD concentration and SNVs for 351 centenarians using quantitative trait association analysis with the PLINK program (version 1.90) adjusted for sex^38^. The genomic inflation est. lambda (based on median chisq) was 1.029, indicating that no obvious inflation occurred. Manhattan plot was created using qqman package for R script^39^. An enlarged view of a Manhattan plot with recombination rate information was generated using LocusZoom^40^.

### Genotyping of *SOD3* p.R231G (rs1799895 C > G)

To determine the *SOD3* genotype against all Very Old and Centenarians, we genotyped an SNV corresponding to *SOD3* p.R231G (rs1799895 C > G) using the TaqMan SNP Genotyping Assays system according to the manufacturer’s standard protocols.

### Statistical analysis

Baseline characteristics, medical history, biomarkers in plasma, and genotype/sequencing are expressed as median or number with percentage or interquartile range (IQR).The differences in plasma EC-SOD concentration distributions were evaluated using the Wilcoxon rank-sum test or ANOVA. Associations between plasma EC-SOD concentration and medical history were analyzed with the generalized linear model using the glm function with or without adjustment for age, sex, and educational history. All statistical analyses were performed using the R script (version 4.0.3) with glmnet (version 4.0), exactRankTests (version 0.8-31), and default packages^41^.

## References

[1] Lopez-Otin C, Blasco MA, Partridge L, Serrano M, Kroemer G (2013). The hallmarks of aging. Cell.

[2] Flatt T (2012). A new definition of aging?. Front. Genet..

[3] Harman D (1956). Aging: a theory based on free radical and radiation chemistry. J. Gerontol..

[4] McCord JM, Fridovich I (1969). Superoxide dismutase. An enzymic function for erythrocuprein (hemocuprein). J. Biol. Chem..

[5] Zelko IN, Mariani TJ, Folz RJ (2002). Superoxide dismutase multigene family: a comparison of the CuZn-SOD (SOD1), Mn-SOD (SOD2), and EC-SOD (SOD3) gene structures, evolution, and expression. Free Radic. Biol. Med..

[6] Marklund SL (1990). Expression of extracellular superoxide dismutase by human cell lines. Biochem. J..

[7] Adachi T, Marklund SL (1989). Interactions between human extracellular superoxide dismutase C and sulfated polysaccharides. J. Biol. Chem..

[8] Adachi T (1996). Substitution of glycine for arginine-213 in extracellular-superoxide dismutase impairs affinity for heparin and endothelial cell surface. Biochem. J..

[9] Yamada H (1995). Molecular analysis of extracellular-superoxide dismutase gene associated with high level in serum. Jpn. J. Hum. Genet..

[10] Sandstrom J, Nilsson P, Karlsson K, Marklund SL (1994). 10-fold increase in human plasma extracellular superoxide dismutase content caused by a mutation in heparin-binding domain. J. Biol. Chem..

[11] Rosen DR (1993). Mutations in Cu/Zn superoxide dismutase gene are associated with familial amyotrophic lateral sclerosis. Nature.

[12] Juul K (2004). Genetically reduced antioxidative protection and increased ischemic heart disease risk: The Copenhagen City Heart Study. Circulation.

[13] Adachi T, Inoue M, Hara H, Maehata E, Suzuki S (2004). Relationship of plasma extracellular-superoxide dismutase level with insulin resistance in type 2 diabetic patients. J. Endocrinol..

[14] Strom A (2017). Lower serum extracellular superoxide dismutase levels are associated with polyneuropathy in recent-onset diabetes. Exp. Mol. Med..

[15] Kobylecki CJ, Afzal S, Nordestgaard BG (2015). Genetically low antioxidant protection and risk of cardiovascular disease and heart failure in diabetic subjects. EBioMedicine.

[16] Mohammedi K (2015). Plasma extracellular superoxide dismutase concentration, allelic variations in the SOD3 gene and risk of myocardial infarction and all-cause mortality in people with type 1 and type 2 diabetes. Cardiovasc. Diabetol..

[17] Juul K, Tybjaerg-Hansen A, Marklund S, Lange P, Nordestgaard BG (2006). Genetically increased antioxidative protection and decreased chronic obstructive pulmonary disease. Am. J. Respir. Crit. Care Med..

[18] Young RP (2006). Functional variants of antioxidant genes in smokers with COPD and in those with normal lung function. Thorax.

[19] Siedlinski M, van Diemen CC, Postma DS, Vonk JM, Boezen HM (2009). Superoxide dismutases, lung function and bronchial responsiveness in a general population. Eur. Respir. J..

[20] Gondo Y (2006). Functional status of centenarians in Tokyo, Japan: developing better phenotypes of exceptional longevity. J. Gerontol. A Biol. Sci. Med. Sci..

[21] Takayama M (2007). Morbidity of Tokyo-area centenarians and its relationship to functional status. J. Gerontol. A Biol. Sci. Med. Sci..

[22] Arai Y (2014). Physical independence and mortality at the extreme limit of life span: Supercentenarians study in Japan. J. Gerontol. A Biol. Sci. Med. Sci..

[23] Arai Y (2015). Inflammation, but not telomere length, predicts successful ageing at extreme old age: a longitudinal study of semi-supercentenarians. EBioMedicine.

[24] Arai Y (2010). The Tokyo Oldest Old survey on Total Health (TOOTH): A longitudinal cohort study of multidimensional components of health and well-being. BMC Geriatr..

[25] Arita Y (1999). Paradoxical decrease of an adipose-specific protein, adiponectin, in obesity. Biochem. Biophys. Res. Commun..

[26] Hotta K (2000). Plasma concentrations of a novel, adipose-specific protein, adiponectin, in type 2 diabetic patients. Arterioscler. Thromb. Vasc. Biol..

[27] Gao D (2020). SOD3 is secreted by adipocytes and mitigates high-fat diet-induced obesity, inflammation, and insulin resistance. Antioxid. Redox. Signal.

[28] Davey A (2012). Diabetes mellitus in centenarians. J. Am. Geriatr. Soc..

[29] Tan RJ (2015). Extracellular superoxide dismutase protects against proteinuric kidney disease. J. Am. Soc. Nephrol..

[30] Hartney JM (2014). A common polymorphism in extracellular superoxide dismutase affects cardiopulmonary disease risk by altering protein distribution. Circ. Cardiovasc. Genet..

[31] Olsen DA (2004). The intracellular proteolytic processing of extracellular superoxide dismutase (EC-SOD) is a two-step event. J. Biol. Chem..

[32] Petersen SV (2005). The high concentration of Arg213–>Gly extracellular superoxide dismutase (EC-SOD) in plasma is caused by a reduction of both heparin and collagen affinities. Biochem. J..

[33] Kwon MJ, Lee KY, Lee HW, Kim JH, Kim TY (2015). SOD3 variant, R213G, altered SOD3 function, leading to ROS-mediated inflammation and damage in multiple organs of premature aging mice. Antioxid. Redox Signal.

[34] Tadaka S (2019). 3.5KJPNv2: An allele frequency panel of 3552 Japanese individuals including the X chromosome. Hum. Genome Var..

[35] Hirata T (2020). Associations of cardiovascular biomarkers and plasma albumin with exceptional survival to the highest ages. Nat. Commun..

[36] Li H, Durbin R (2009). Fast and accurate short read alignment with Burrows–Wheeler transform. Bioinformatics.

[37] DePristo MA (2011). A framework for variation discovery and genotyping using next-generation DNA sequencing data. Nat. Genet..

[38] Chang CC (2015). Second-generation PLINK: Rising to the challenge of larger and richer datasets. Gigascience.

[39] Turner SD (2018). qqman: An R package for visualizing GWAS results using Q-Q and manhattan plots. J. Open Source Softw..

[40] Pruim RJ (2010). LocusZoom: Regional visualization of genome-wide association scan results. Bioinformatics.

[41] R Core Team. R. A language and environment for statistical computing (R Foundation for Statistical Computing, Vienna, 2020).

